# A154 TEMPORAL TRENDS OF DEPRESSION HOSPITALIZATION AND SUICIDE DEATH IN THOSE WITH INFLAMMATORY BOWEL DISEASE

**DOI:** 10.1093/jcag/gwae059.154

**Published:** 2025-02-10

**Authors:** A Markovinovic, S Coward, C Ma, A Shaheen, G G Kaplan

**Affiliations:** University of Calgary, Calgary, AB, Canada; University of Calgary, Calgary, AB, Canada; University of Calgary, Calgary, AB, Canada; University of Calgary, Calgary, AB, Canada; University of Calgary, Calgary, AB, Canada

## Abstract

**Background:**

Individuals with inflammatory bowel disease (IBD) are known to be more susceptible to mental health disorders such as depression. However, literature examining severe outcomes of depression in IBD, including hospitalizations due to depression and death by suicide, is limited.

**Aims:**

To describe trends in depression hospitalizations and suicide deaths in individuals with IBD living in Alberta compared to matched controls.

**Methods:**

This population-based study used a validated algorithm in administrative data to identify the prevalent IBD population and 10 to 1 age- and sex-matched controls in Alberta from fiscal year 2002/03 to 2021/22. The primary outcomes were hospitalization for depression and suicide death. Annual prevalence rates of depression hospitalization were calculated per 1,000 persons. Average annual percent change (AAPC) with 95% confidence intervals (CI) were calculated from Poisson or negative binomial models. Rates and AAPCs for depression hospitalization in IBD cases were stratified by age (<18, 18–40, 41–64, and ≥65), sex (male or female), and IBD type (Crohn’s disease or ulcerative colitis and inflammatory bowel disease unclassified [IBD-U]). Suicide deaths were reported as a proportion of the prevalent IBD population and as an AAPC (95% CI) calculated from a log-binomial model.

**Results:**

Table 1 describes depression hospitalizations in the prevalent IBD population (*n*=42,903). Hospitalization rates for depression in those with IBD decreased with an AAPC of −4.25% (95% CI: −5.51, −2.96) from 4.42 per 1,000 in 2002/23 to 2.08 per 1,000 in 2021/22, while hospitalization rates for depression in controls decreased with an APPC of −2.37% (95% CI: −2.90, −1.94) (Table 1, Figure 1). Hospitalization rates for depression in those with IBD decreased for all stratifications except pediatric cases (AAPC: 2.03%; 95% CI: −5.99, 10.72) (Table 1). Suicide deaths occured in 0.23% (*n*=99) of the prevalent IBD population and remained stable over time (AAPC: −0.09%; 95% CI: −3.82, 3.79).

**Conclusions:**

Despite the decreasing hospitalization rates for depression in individuals with IBD, suicide deaths have remained stable at 0.23%.

AAPCs and rates of depression hospitalization in those with IBD, stratified by age, sex, and IBD type

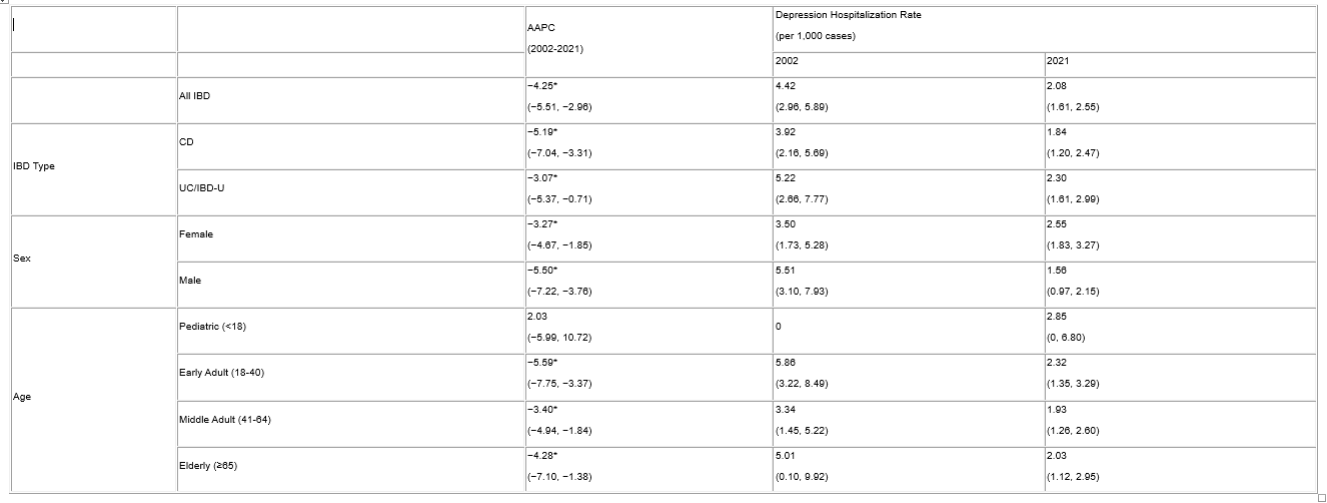

*denotes statistical significance

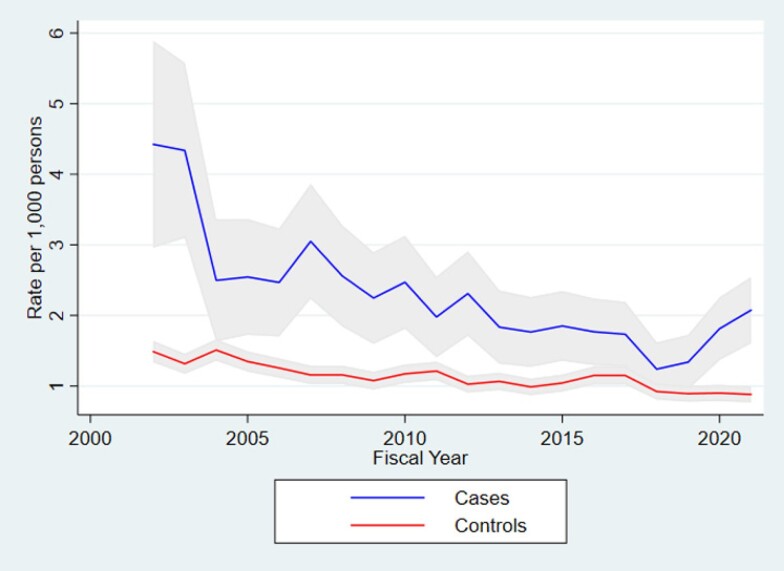

Temporal trends in depression hospitalization in IBD cases and matched controls

**Funding Agencies:**

CIHR

